# Glutathione-related antioxidant defense system in patients with hypertensive retinopathy

**DOI:** 10.22336/rjo.2021.9

**Published:** 2021

**Authors:** Ecaterina Pavlovschi, Valeriana Pantea, Djina Borovic, Olga Tagadiuc

**Affiliations:** * Department of Biochemistry and Clinical Biochemistry, “Nicolae Testemițanu” State University of Medicine and Pharmacy, Chișinău, Republic of Moldova; ** Laboratory of Biochemistry, “Nicolae Testemițanu” State University of Medicine and Pharmacy, Chișinău, Republic of Moldova; *** Ovisus Medical Private Center, Chișinău, Republic of Moldova

**Keywords:** reduced glutathione, glutathione peroxidase, glutathione reductase, antioxidant defense system, hypertensive retinopathy

## Abstract

**Objective:** To analyze the glutathione antioxidant defense system changes in the tear and serum of patients with hypertensive retinopathy (HR) and to establish whether there is an interdependence between their levels and HR degree.

**Methods:** 90 patients were split into three groups according to the Keith-Wagner-Barker grading of HR: GI–36 patients; GII–35 patients; GIII–19 patients. The concentration of reduced glutathione (GSH) and activities of glutathione peroxidase (GPx) and glutathione reductase (GR) in tear and serum were measured. Results were analyzed by ANOVA, followed by Bonferroni *post hoc* test. The Spearman correlation coefficient was calculated (p≤0.05 statistically significant).

**Results:** In serum, the GSH level and GPx activity were not statistically changed between groups with HR degree advancement, unlike the GR activity that was statistically diminished (p=0.018). The values of the studied markers in the tear showed a decrease with the progression of the HR degree. Only serum GSH level correlated with the tear one (r=-0.361, p=0.000), while the enzymes activity did not. A correlation of GPx and GR activity (r=0.417, p=0.000) was identified in tear, while in serum - of GPx activity and GSH level (r=409, p=0.000). Tear GPx and GR levels correlated significantly but with low power with HR degree (r=0.299, p=0.004/ r=0.299, p=0.004).

**Conclusion:** Statistically significant elevation in tear GPx and GR activity and a tendency of GSH level increase was revealed, being attested, and a direct correlation between GPx and GR activity, as well as of their activity with the HR degree. In serum, the GSH level and the GPx activity did not change accurately, while the GR activity diminished significantly, the identified decrease being correlated with the HR degree.

**Abbreviations:** HR = hypertensive retinopathy, HTN = hypertension, GSH = reduced glutathione, GPx = glutathione peroxidase, GR = glutathione reductase, GGR = gamma-glutamyl transferase, ROSs = reactive species of oxygen, OS = oxidative stress

## Introduction

Enhanced blood pressure is a highly frequent risk factor for cardiovascular disease morbidity and mortality. The interest for hypertensive retinopathy (HR) - its major ocular complication comes newly to life, motivated and boosted by the fact that the severity and duration of hypertension (HTN) are directly proportional to the incidence of HR [**1**-**3**]. HR is known as a succession of retinal microvascular transformations generated by augmented uncontrolled blood pressure [**1**,**4**-**6**]. 

The dexterity and experience of the ophthalmologist is nowadays aided in diagnostic by the image techniques, but neither of them can fully estimate the pathological metabolic changes at the level of the retina caused by HTN. In such a way, biochemical markers with known mechanism of involvement in the pathogenesis of the disease, are required as an additional help in the assessment of quantitative and qualitative retinal changes.

As it has been lately proved, HR is a many-sided disorder. Singularly elevated blood pressure does not fully reproduce the expansion of retinopathy, suggesting the existence of extra predisposing and causative factors. If we were to exclude essential and secondary HTN, a multitude of trigger and causing factors to the development of HR were incriminated, among which the following may be listed: inflammation (with an enhanced high-sensitivity C-reactive protein amount), oxidative stress (with augmented serum ferritin and gamma-glutamyl transferase levels), endothelial dysfunction (with a high von Willebrand factor level), stimulated angiogenesis (with decreased adiponectin and increased leptin levels), plus such individual factors as low birth weight, high body mass index, alcohol consumption, etc. [**7**-**14**]. 

Oxidative stress (OS) is still not sufficiently analyzed, even if it is seen as the HR most plausible cause and is known to induce profound changes of different biological structures and molecules, as cellular membranes, lipids, proteins, and nucleic acids. The retina is a highly metabolic organ with a complex 10-layer structure that works entirely on aerobic respiration, consuming the highest quantity of oxygen of all tissues. Due to this, a variety of already well-known reactive species of oxygen (ROSs) such as superoxide anion radical (O2−∙), hydroxyl radical (•OH) and hydrogen peroxide (H2O2), that are extremely harmful and reactive, are generated in retina [**15**,**16**]. 

Retina is constantly exposed to persistent OS through different mechanisms, among them being the regular exposure to light and ROSs, provoked by visual signal transduction pathways, caused by enhanced oxygen consumption, oxidation of polyunsaturated fatty acids and phagocytosis of photoreceptor cells. Normally, every single cell type in the retina is capable to preserve homeostasis under conditions of OS. Despite this, the moment the equilibrium between pro- and antioxidative signaling is disturbed, excessive OS contributes to functional dysregulation and changes that lead to visual deterioration [**17**]. So, in order to survive, the retinal cells request a balance between oxygen, ROSs, and antioxidant molecules that counteract OS destruction.

Hypoxia or any other disbalances in mitochondrial function, caused by HTN, are the main conceivable cause of the OS in HR. Disregarding the manner in which HTN evolves, ROSs still remain a major and fundamental element in the pathogenesis of HR. Persistently enhanced levels of HTN generate an elevation in ROSs, that, in consequence, break the vulnerable balance in the retina, inclining to cytotoxicity and tissue damage, that are observed at fundoscopy by the ophthalmologist and allow the grading of the qualitative extent and progression of HR.

Until present, only two markers of OS have been studied in order to explain the pathological molecular mechanism of HR. Gamma-glutamyl transferase (GGT) and ferritin levels in serum showed a noticeable increase along with the evolution of HR and presented a positive correlation with its grade [**9**,**10**]. 

Under physiological conditions, the harmful effects of ROSs can be kept under control by specific enzymes and low molecular weight substances that eradicate ROSs and contribute to the redox balance in the cell [**3**]. Glutathione and the related enzymes are a part of the defense system protecting the eye against chemical and OS. 

Reduced glutathione (GSH), a major thiol-disulfide redox cell buffer, is one of the most conclusive scavengers of ROS that might be used as a parameter of antioxidant system. Extensive research during the past half century have shown the presence of glutathione in all cells. The synthesis of GSH from its constituent amino acids requires ATP and it is catalyzed by the enzyme γ-glutamyl cysteine synthetase and GSH synthetase. Transcription and activity of γ-glutamyl cysteine synthetase is increased by several factors and processes, among which the following can be mentioned: depletion or conjugation of GSH, inflammatory cytokines, antioxidants, nitrosative stress and OS [**18**]. The diminished synthesis of GSH, along with its use in detoxification, the augmented breakdown, or the failure to regenerate GSH from the oxidized form GSSG persuades decreased GSH levels, being partly responsible for oxidative stress, which is implicated in the pathogenesis of a series of diseases [**19**]. OS is correlated with depletion and oxidation of GSH, that was noted in hypertensive subjects when compared with non-hypertensive ones [**20**,**21**]. 

Glutathione-dependent enzymes like glutathione peroxidase (GPx) and glutathione reductase (GR), along with NADPH producing enzymes: glucose-6-phosphate dehydrogenase and ICDH-NADPH depending, belong to the defense mechanism against oxidation. GSH, GR and GPx are proven to be in different types of retina cells [**22**], that will be a part of the complex protective eye defense mechanism against OS.

There are many factors that can ultimately induce an increased susceptibility of the retina to oxidation, among which is light, polyunsaturated fatty acids, high oxygen flux, etc. The overcoming of the protective factors leads to the retinal changes visualized at fundoscopy. The retinal sensitivity to OS implies the fact that many retinal diseases involve the oxidative damage of the molecules and cells, this being a motivating factor to study this phenomenon in HR.

The above-mentioned information confirms that oxidative stress and the inability to antioxidant protection are confirmed pathogenic mechanisms of many diseases, including HTN. At the same time, there are no studies that would certify for sure the involvement of the OS/ antioxidant system imbalance in general and the changes of the GSH system in the HR appearance and progression. We did not identify studies in the specialty literature and on the correlation of changes in the level of markers stated in tear and serum, which would establish the possibility of their use in the diagnosis and monitoring of HR.

The aim of the study was to analyze the glutathione antioxidant defense system in the tear and serum of patients with hypertensive retinopathy and to establish whether there is an interdependence between their levels and grade of HR.

## Material and methods 

***Study design***

The study was approved by the Research Ethics Committee of the “Nicolae Testemițanu” State University of Medicine and Pharmacy, Chișinău, Republic of Moldova (12.02.2018). A written informed consent was obtained before the inclusion in the research. All the procedures performed in studies involving human participants were in accordance with the ethical standards of the institutional and/ or national research committee and with the 1964 Helsinki declaration and its later amendments or comparable ethical standards.

90 hypertensive patients, among which 38 males (42.2%) and 52 females (57.8%), with a mean age of 59.79 ± 12.29 years (range: 38‒88) were split into three groups according to the Keith-Wagner-Barker grading systems for HR based on fundus examination, as it follows: group 1 (GI): 36 patients with 1st grade of HR - with mild generalized retinal arteriolar narrowing; group 2 (GII): 35 patients with 2nd grade of HR - with definite focal narrowing and arteriovenous nipping; group 3 (GIII): 19 patients with 3rd grade of HR - with signs of grade 2 retinopathy and retinal hemorrhages, exudates and cotton wool spots. No patients with 4th grade of HR - with severe grade 3 retinopathy and papilledema were involved in the study, due to their insufficient number and association of additional pathologies [**2**,**23**].

***Patient selection***

Hypertensive patients, who came for an examination at Ovisus Medical Center between 2018 and 2019 and in whom the diagnosis of HR was primarily confirmed, established after a comprehensive specialized ophthalmological consult with the determination of visual acuity, autorefractor-keratometry, perimetry, anterior and fundus biomicroscopy, ultrasonography, tonometry, gonioscopy, optical coherence tomography (OCT) of the macular area and the papilla of the optic nerve. 

At the time of samples collection, the patients were not on any antihypertensive or other medication that could interfere with the results of the research. Moreover, the patients with metabolic disorders like diabetes and severe obesity, serious somatic comorbidities, with renal and neurological pathologies, ocular trauma, optic nerve atrophies of various causes and ocular associated diseases such as glaucoma, diabetic retinopathy, acute and chronic inflammatory processes, and uveitis, were excluded from the study.

***Sample collection***

Samples of venous blood (5 ml) were collected, centrifuged and serum was separated. Tear samples were collected with microcapillary tubes from the inner tear lake of the lateral conjunctival sac of the inferior fornix. Serum and tear were dispensed into Eppendorf microtubes and frozen (-40ºC) in expectation of being tested.

***Biochemical analysis***

Determination of serum and tear reduced glutathione was performed according to the nitroprusside method described by Mortensen E in the modification of Andronache L et al. [**24**,**25**]. The principle of the method is based on the interaction of reduced glutathione in an alkaline environment with nitroprusside and cyan ions with the formation of a complex red-violet compound, the intensity of the color being directly proportional to the GSH concentration in the investigated biological material. GSH quantity is reported in µM/ L for both serum and tear samples.

Serum and tear glutathione reductase activity assay was performed according to the procedure described by Smith JW et al. modified by the Gudumac V et al., which is based on the determination of the rate of increase of the reduced glutathione level (GSH) in the enzymatic reaction. GSH was measured in the reaction with 5,5'-dithiobis-2-nitrobenzoate, the reaction product (2-nitro-5-benzoate) being determined photometrically at 405 nm [**26**,**27**]. The activity of the enzyme is presented in nmol/ s·L.

The dosing of glutathione peroxidase activity in the biological material was performed based on the Warburg optical test, according to the procedure described by Wendel A modified by Tagadiuc O et al. [**28**]. The principle of this process is based on the determination of the rate of decrease in the level of reduced glutathione (GSH) in the reaction media for which 5,5'-dithiobis (2-nitrobenzoate) is used, which reacts with GSH. The reaction product (2-nitro-5-thiobenzoate) is determined photometrically at 405 nm. GPO activity was expressed nmol/ s·L. 

***Statistical analysis***

The null hypothesis that the mean values of analyzed enzymes are the same across all the categories of HR, was tested. The data obtained were processed using SPSS 23.0 Software. The values of studied markers were presented as mean ± SD and analyzed by applying ANOVA followed by the Bonferroni *post hoc* test. Correlation analysis was performed using Spearman correlation test. Differences were considered statistically significant if the two-tailed p value was 0.05 or less.

## Results

**Fig. 1:**
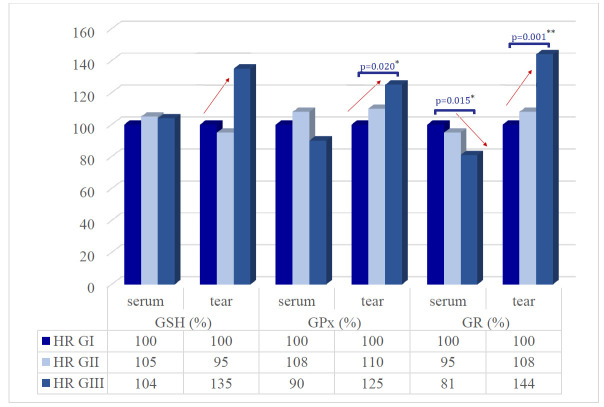
GSH content and glutathione peroxidase (GPx) and glutathione reductase (GR) activities in serum and tear of the patients with different grade of HR

In order to examine glutathione antioxidant defense system in patients with hypertensive retinopathy, we investigated the concentration of reduced glutathione (GSH) and activities of the main antioxidant enzymes implicated in metabolism of glutathione - glutathione peroxidase (GPx) and glutathione reductase (GR). The research results are summarized in the data in **Fig. 1**.

The tear samples of all three researched markers showed promising results, ultimately an increase in values as HR advanced in grade being observed. 

A tendency of statistically insignificant fluctuation of reduced glutathione (GSH) levels between groups (p=0.357) was observed in the tear, being diminished in GII in comparison with GI (-5%; 152,94 ± 68.81 µM/ L vs. 160.81 ± 59.87 µM/ L, p=0.141), and elevated in GIII compared to GII (+40%; 217.18 ± 175.41 µM/ L vs. 152,94 ± 68.81 µM/ L, p=1.00). 

In the tear, GPx values were 288.40 ± 80.04 nM/ s·L in the Ist group, 317.14 ± 99.82 nM/ s·L (+10%, p=0.574) in the IInd group and 360.68 ± 97.37 nM/ s·L (+15%, p=0.299) in the IIIrd group of patients with HR. A statistically significant difference was observed between group I - 288.40 ± 80.04 nM/ s·L and III - 360.68 ± 97.37 nM/ s·L (p=0.020). Moreover, a statistically significant fluctuation of GPx levels between groups in tear (p=0.025) as the HR progressed was attested.

GR activity presented the same statistically significant differences between groups such as GPx, with an increase in tear sample (p=0.001) along with the evolution of HR, being boosted in GII in comparison with GI (+8%; 160,09 ± 55.05 nM/ s·L vs. 148.96 ± 44.34 nM/ s·L, p=1.00), and elevated in GIII compared to GII (+36%; 215.10 ± 100.16 nM/ s·L vs. 160,09 ± 55.05 nM/ s·L, p=0.01). In addition, a statistically significant change was observed between GIII - 215.10 ± 100.16 nM/ s·L and GI - 148.96 ± 44.34 nM/ s·L, p = 0.001.

In serum, the GSH and GPx values were not statistically changed between groups, unlike GR that was statistically diminished.

A statistically insignificant fluctuation of GSH levels in serum was attested between groups (p=0.926) as HR progressed. In paired group comparisons, the serum GSH level in GII increased compared to GI (+5%; 1785.42 ± 491.22 µM/ L vs. 1703.94 ± 533.02 µM/ L, p=1.0), but decreased in GIII compared to GII (-1%; 1763.02 ± 483.46 µM/ L vs. 1785.42 ± 491.22 µM/ L, p=1.0) (**Fig. 1**). 

Moreover, a statistically insignificant fluctuation of GPx activity in serum (p=0.223) was attested through the evolution in grade of the retinopathy. GPx serum level in GII increased compared to GI (+ 8%; 476.05 ± 154.75 nM/ s L vs. 441.62 ± 147,04 nM/ s L, p = 1.0), diminished in GIII compared to GII (- 18%; 399.15 ± 178.58 nM/ s L vs. 476.05 ± 154.75 nM/ s L, p = 0.268) (**Fig. 1**). 

Unlike GSH and GPx, serum GR activity presented a statistically significant different picture between groups, with a decrease activity (p=0.018). Being more specific, in paired group comparisons, the serum GR level in GII diminished compared to GI (-5%; 647.68 ± 190.54 nM/ s L vs. 685.26 ± 139.62 nM/ s L, p=0.997), and in GIII compared to GII (-14%; 552.46 ± 144.97 nM/ s L vs. 647.68 ± 190.54 nM/ s L, p=0.128) (**Fig. 1**). A statistically significant modification was seen in GIII - 552.46 ± 144.97 nM/ s L compared to GI - 685.26 ± 139.62 nM/ s L (p = 0.015).

The correlation between the serum and tear indexes is shown in **Table 1**.

**Tabel 1:** Correlation of GSH, GPx and GR levels among them in serum and tear

		GSH		GPx		GR	
		in serum	in tear	in serum	in tear	in serum	in tear
GSH	in serum	1.000	-0.361/ p=0.000	0.409/ p=0.000	-0.041/ p=0.705	0.202/ p=0.056	-0.129/ p=0.227
	in tear	-0.361/ p=0.000	1.000	-0.197/ p=0.063	0.024/ p=0.821	-0.173/ p=0.102	0.190/ p=0.073
GPx	in serum	0.409/ p=0.000	-0.197/ 0.063	1.000	-0.170/ p=0.109	0.187/ p=0.077	-0.116 /p=0275
	in tear	-0.041/ p=0.705	0.024/ p=0.821	-0.170/ p=0.109	1.000	-0.094/ p=0.381	0.417/ p=0.000
GR	in serum	0.202/ p=0.056	-0.173/ p=0.102	0.187/ p=0.077	-0.094/ p=0.381	1.000	-0.039/ p=0.714
	in tear	-0.129/ p=0.227	0.190/ p=0.073	-0.116/ p=0.275	0.417/ p=0.000	-0.039/ p=0.714	1.000

A significant weak negative correlation was established between tear and serum GSH levels (r=-0.361, p=0.000), while no correlation between tear and serum GPx (r=-0.170, p=0.109) and GR (r=-0.039/p=0.714) activities was established.

We also analyzed a feasible association of the modification in GSH level with the activity of enzymes, which consume GSH as a cofactor for their detoxification and antioxidant activity. After the evaluation of the data (**Table 1**), we were able to underline some potential correlation, beside those mentioned before, between the following markers: tear GPO and tear GR (r=0.417, p=0.000) and serum GSH and serum GPO (r=0.409, p=0.000), which presented medium strength positive and significant results. 

In both researched fluids, the GSH level did not correlate with the degree of HR (r=-0.008, p=0.941 in serum/ r=0.039, p=0.716 in tear). GPx activity in serum did not show a correlation with the grade of HR (r = -0.053; p = 0.621), while the tear GPx had a significant weak strength, positive correlation with the grade of HR (r=0.299, p=0.004). In both researched fluids, GR activity showed a significantly weak and positive correlation with the grade of HR (r=0,297, p=0.004 in serum/ r=0.252, p=0.017 in tear) (**Table 2**).

**Tabel 2:** Correlation of GSH, GPx and GR levels among them in serum and tear

		GSH		GPx		GR	
		in serum	in tear	in serum	in tear	in serum	in tear
Retinopathy	Correlation coefficient	-0.008	0.039	-0.053	0,299	0,297	0.252
	Statistical significance, 2-tailed (p)	0.941	0.716	0.621	0.004	0.004	0.017

## Discussion

HR associated with HTN is a multifaced pathology. OS with the inadequate antioxidant defense is one of the possible mechanisms of HR development in HTN. This way, the investigation of glutathione antioxidant defense system in patients with HR is pivotal for the role establishment of the OS/ antioxidant defense disequilibrium in the pathogenesis of HR.

Up to now, much of the research regarding OS, the antioxidant system, and the glutathione system in HTN, have been performed in serum. The latest studies emphasized reduced serum GSH levels in diseases such as Parkinson, age-related diseases, extracted mature cataract, etc. [**29**-**31**]. A diminished serum GSH concentration was found in patients with acute myocardial infarction and has been considered the predictor of coronary restenosis after percutaneous coronary intervention [**21**,**31**]. Decrease serum GSH concentration may also lead to a lower GPx activity, explained by the fact that GSH is one of its substrates [**32**]. 

The researches of the past decade showed that HTN is correlated with deteriorations in glutathione metabolism. Chaves et al. identified a significantly lower GSH and GPx values in HTN patients in comparison with the control group, explaining this by impaired enzyme expression response and enzyme inactivation due to OS. Antihypertensive treatment that decreases OS, results in an increase of GSH [**33**]. Moreover, Silva et al. identified a decrease in the activity of GR in HTN [**34**]. Induced HTN may be associated with enhanced superoxide radical level, as it was pointed in some researches [**19**,**35**]. All things considered, it is implied that OS with alterations in glutathione metabolism as a result, is strongly related to the pathogenesis of HTN [**36**]. 

To conclude, the appearance of OS might be either a repercussion of a decline in the antioxidant defense system activity or an augmentation of ROS concentration. Therefore, the relationship between blood pressure and OS markers is still not clarified [**19**,**35**]. 

HTN comprises various and complex biochemical pathways, nevertheless OS and nitrosative stress are considered the main ROSs sources [**36**,**37**]. A boost in ROS generation in the wall of the vessels leads to endothelial dysfunction and vascular inflammation [**38**,**39**]. Accentuated ROSs values trigger the growth of vascular smooth muscular cells, enhanced contractility, monocytes incursion, peroxidation of the lipids, inflammation, and increased deposition of extracellular matrix proteins. All mentioned are pivotal factors in hypertensive vascular injury and lead to the development of the clinical signs visualized at fundoscopy by an ophthalmologist [**38**,**40**-**42**]. 

Rybka J et al. analyzed glutathione antioxidant defense system in elderly patients treated for HTN. They measured the concentration of glutathione in whole blood and activities of glutathione peroxidase, glutathione transferase, and glutathione reductase in erythrocytes, noticing an enhancement in the mean activity of all researched enzymes that were significantly higher in HTN compared with the control group. These results suggested the importance of glutathione system in blood pressure regulation [**3**]. 

Rybka J et al. study did not involve the evaluation of HR, tear markers and the assessment of serum - tear correlations. In our study, as in this research for the 2nd group, we obtained a similar enhancement of the GSH and GPX values in serum, followed unexpectedly by a decrease in the third group, plus gradually lowered value of serum GR. The relation between GSH concentration and GR activity was not as expected, namely these two markers were not significantly correlated, which for sure suggested HTN-related disturbances in glutathione defense system. These changes could be explained as a cell defense mechanism facing an augmented OS. Consequently, we might suggest that the assumption of OS involvement in the development of HR incipience was justified.

The association of OS with the outcome of HR was concretely highlighted solely in few recent studies. As it was mentioned earlier, merely two OS serum markers have been investigated by present. Karaca et al. pointed out an augmented level of γ-glutamyl transferase activity in HR, which is a polyfunctional enzyme considered to have a crucial role in glutathione homeostasis needed to preserve enough concentrations of intracellular glutathione and able to protect the cells against oxidants [**9**]. The second marker studied by Coban et al. highlighted the correlation between HR and high ferritin level in serum, that might be linked to an increase level of OS due to iron involvement in Fenton reaction [**10**]. 

Our results brought something new because the enzymes’ activities were tested in the tear, these analyses being more informative in comparison with the serum ones, which reflect the general injury of the organism in HTN. 

The changes in the tear are very logical: a) in the 2nd degree of HR, the GSH level diminished, because of the enhancement in GPx activity, which is a consequence of an increased OS, but the augmentation of GR it is not sufficient for the GSH restoration; b) in the 3rd degree, an increase in GSH level was observed, because the enhancement in GPx activity is compensated by an augmentation in GR activity, and we also remarked a correlation between GPx and GR in tear. Thus, it can be briefly concluded that the antioxidant protection system of GSH is increasing its capacity along the progression of HR, which attests 2 things: a) the amplification of OS simultaneously with the increase of the retinal damage; b) the major defensive role of the GSH system in the late stages. 

There are almost no correlations between tear and serum markers, so serum indices do not reveal ocular changes and therefore they cannot be used as markers of HR assessment.

The results are interesting and reveal the need for further studies by evaluating other factors involved in GSH metabolism and the functioning of the GSH system, as well as other constituents of the antioxidant system, such as SOD, catalase, vitamin A, micromolecular thiols, etc.

## Study limitations

Nonetheless, this research had several limitations, questioned due to the changes of the GSH, GR and GPx during grades of retinopathy, that cannot entirely be elucidated only by HTN, HR or both synchronously. Moreover, our results could not define if the diminished enzymes levels in serum and amplificated in tear predict the development of retinopathy or contrarily is an effect.

Our study was conducted on hypertensive patients who were not on any antihypertensive medication at that moment or any other treatment that could discredit the results and could not be applied on patients under treatment.

An expanse of the study group is needed in order to ensure more reliable results that can be applied as a prognostic and diagnostic method. Also, we must be careful in generalizing the conclusions related to the fact that the study was made only in one hospital, but its expansion across different hospitals and countries will be necessary.

## Conclusions

Our study completes the existing knowledge on the disorders of metabolic processes at the level of the eye in HTN, which conditions the development of retinopathy.

The advantage of the study is the evaluation of GSH system indices simultaneously in tear and serum, in patients with different degrees of HR, who were not under antihypertensive or other treatment, which allowed the obtaining of “pure” data, unaltered by other factors.

It has been established that there is a progressive increase in the activity of the GSH system, manifested by an increased GSH content and GPx and GR activities in tears, serum changes being of opposite direction and basically lacking sero-lacrimal correlations.

More studies are needed to ultimately clarify the role of the OS and glutathione-related antioxidant defense system in the HR development and to ascertain the threshold values of the mentioned enzymes, with their potential use in the patient stratification in groups and a better grading system that will reflect the clinical modification seen on the fundoscopy of the retina.

**Conflict of Interest**

The authors declare no conflict of interest.

**Informed Consent and Human and Animal Rights statements**

Informed consent has been obtained from all individuals included in this study.

**Authorization for the use of human subjects**

Ethical approval: The research related to human use complies with all the relevant national regulations, institutional policies, is in accordance with the tenets of the Helsinki Declaration, and has been approved by the Ethics Committee of “Nicolae Testemițanu” State University of Medicine and Pharmacy, Chișinău, Republic of Moldova.

**Acknowledgements**

None.

**Sources of Funding**

Doctoral grant offered by the Ministry of Education, Culture and Research of Republic of Moldova.

**Disclosures**

None of the authors has any financial interest to disclosure. 
